# Diagnostic Performance of Triglyceride-Glucose Body Mass Index (TyG-BMI), Fibrosis-4 Index (FIB-4), and Their Combined Model for Clinically Significant Liver Fibrosis in Type 2 Diabetes Mellitus

**DOI:** 10.7759/cureus.117491

**Published:** 2026-10-05

**Authors:** Tahseer Khan, Syeda Batool Tirmizi, Aamir Shehzad, Arslan Shamim, Zara Ramzan, Ahsan Raza, Sarah Khan

**Affiliations:** 1 Internal Medicine, Dow University Hospital, Karachi, PAK; 2 Gastroenterology, Lahore General Hospital, Lahore, Lahore, PAK; 3 Internal Medicine, Ghurki Trust Teaching Hospital, Lahore, PAK; 4 Nephrology, Services Hospital Lahore, Lahore, PAK; 5 Emergency Medicine, Government Trauma Centre Lalamusa, Lalamusa, PAK; 6 Acute Medicine, William Harvey Hospital, East Kent Hospitals University Foundation Trust, Ashford, GBR; 7 Internal Medicine, Shaikh Zayed Medical College and Hospital, Rahim Yar Khan, PAK

**Keywords:** liver fibrosis, metabolic dysfunction-associated steatotic liver disease (masld), transient elastography, triglyceride-glucose body mass index, type 2 diabetes mellitus

## Abstract

Introduction

Patients with type 2 diabetes mellitus are at increased risk of clinically significant liver fibrosis, while commonly used noninvasive fibrosis scores do not perform equally well in all patients. The triglyceride-glucose body mass index (TyG-BMI) captures metabolic changes and excess body weight and may add information not included in the fibrosis-4 index (FIB-4). This study assessed how well the TyG-BMI and FIB-4 identified clinically significant liver fibrosis when used alone and together.

Methods

This cross-sectional diagnostic accuracy study included 280 patients with type 2 diabetes mellitus. The TyG-BMI and FIB-4 were calculated using the patients’ clinical and laboratory measurements. Transient elastography served as the reference test, with liver stiffness measurement ≥8 kPa used to identify clinically significant fibrosis. Accuracy was assessed by receiver operating characteristic analysis and by calculating sensitivity, specificity, positive predictive value, negative predictive value, and overall accuracy.

Results

Clinically significant liver fibrosis was present in 66 of 280 patients (23.6%). The TyG-BMI showed moderate discrimination, with an area under the curve (AUC) of 0.710 (95% CI 0.633-0.783), while the FIB-4 showed an AUC of 0.801 (95% CI 0.736-0.857). At a FIB-4 cutoff of 1.30, sensitivity was 78.8%, specificity was 69.2%, and negative predictive value was 91.4%. The combined TyG-BMI-FIB-4 model achieved an AUC of 0.850 (95% CI 0.796-0.901), with 78.8% sensitivity, 79.0% specificity, 53.6% positive predictive value, 92.3% negative predictive value, and 78.9% accuracy. The internally validated AUC was 0.845.

Conclusions

Combining the TyG-BMI with the FIB-4 improved discrimination for transient elastography-defined clinically significant liver fibrosis compared with either index alone. This approach may improve noninvasive fibrosis risk stratification in patients with type 2 diabetes mellitus, but external validation is required before clinical application.

## Introduction

Metabolic dysfunction-associated steatotic liver disease (MASLD) occurs frequently in people with type 2 diabetes mellitus (T2DM), a population recognized by current diabetes guidance as being at increased risk of clinically important hepatic fibrosis [1]. In prospective screening, about two-thirds of adults with T2DM had hepatic steatosis and 14% had advanced fibrosis, indicating that substantial liver disease may be present without prior clinical recognition [2]. Because fibrosis is strongly related to progression toward cirrhosis and liver-related complications, timely risk assessment is relevant in diabetes care [1].

The fibrosis-4 index (FIB-4) is commonly used for initial noninvasive fibrosis assessment and is derived from age, aspartate aminotransferase, alanine aminotransferase, and platelet count. Current guidance advises further assessment with transient elastography when FIB-4 is elevated [1,3]. Its diagnostic performance, however, varies in T2DM. Reported areas under the curve (AUCs) range from 0.901 in an Indian population with clinically significant fibrosis to 0.653 in a Korean diabetic population [3,4]. Reduced accuracy has also been observed when histology was used as the reference method [5].

The triglyceride-glucose body mass index (TyG-BMI) is calculated from fasting triglycerides, fasting glucose, and BMI and therefore represents both metabolic dysfunction and excess adiposity. These characteristics are relevant to MASLD but are not components of FIB-4. Although higher TyG-BMI values have been linked with liver fibrosis, its stand-alone diagnostic ability has generally been moderate [6]. Studies using either histology or elastography have reported AUCs around 0.63-0.70 [7,8]. Differences in glycemic state may also influence its performance, which is particularly important when the index is applied to patients with T2DM [9].

TyG-BMI and FIB-4 are based on different clinical information and therefore do not describe the same part of a patient’s fibrosis risk. TyG-BMI is derived from fasting glucose, triglycerides, and BMI, so it mainly reflects metabolic status and body weight. FIB-4, in contrast, is calculated from age, aminotransferase levels, and platelet count, which are more directly related to liver fibrosis assessment. Because the two scores use different information, looking at them together may give a broader picture than relying on either score alone. However, this particular combination has not been studied extensively, especially in South Asian patients with type 2 diabetes mellitus [10]. For this reason, the present study examined TyG-BMI and FIB-4 separately and then assessed a model that included both scores. Transient elastography was used to determine whether clinically significant liver fibrosis was present.

## Materials and methods

This cross-sectional diagnostic-accuracy study was performed at the Department of Gastroenterology, Mayo Hospital Lahore, Pakistan. This study was performed over a duration of one year from January 2025 to December 2025. Patients with T2DM were enrolled consecutively after providing written informed consent. Approval was obtained from the institutional ethics committee (approval 166/RC/KEMU), and study procedures conformed to the Declaration of Helsinki [11].

Men and women aged 35-70 years with established T2DM were eligible. Diabetes was documented from the medical record or by accepted diagnostic criteria: HbA1c ≥6.5%, fasting plasma glucose ≥126 mg/dL, two-hour plasma glucose ≥200 mg/dL after a 75-g oral glucose load, or random plasma glucose ≥200 mg/dL with typical hyperglycemic symptoms [12]. Exclusion criteria were chronic hepatitis B or C, other established chronic liver disease, significant alcohol use, secondary hepatic steatosis, acute hepatitis, cholestasis, pregnancy, decompensated heart failure, and incomplete or unreliable transient elastography.

Patients’ age, sex, diabetes duration, BMI, waist circumference, systolic and diastolic blood pressure, hypertension, dyslipidemia, and smoking status were recorded on a data collection proforma. Fasting laboratory testing included plasma glucose, HbA1c, triglycerides, total cholesterol, LDL-C, HDL-C, ALT, AST, GGT, and platelet count. The TyG index and FIB-4 were calculated as follows [13-15]:

$\text{TyG index} = \ln\left(\frac{\text{Fasting triglycerides (mg/dL)} \times \text{Fasting glucose (mg/dL)}}{2}\right)$

$\mathrm{TyG-BMI} = \mathrm{TyG} \times \mathrm{BMI}$

$\mathrm{FIB-4} = \frac{\mathrm{Age} \times \mathrm{AST}}{\text{Platelet count} \times \sqrt{\mathrm{ALT}}}$

Thresholds of 1.30 and 2.67 were prespecified for FIB-4. The prespecified FIB-4 thresholds were applied in the main analysis; a post hoc subgroup analysis examined FIB-4 ≥1.30 and an age-adjusted threshold of ≥2.00 among participants older than 65 years [1].

Transient elastography was performed with FibroScan (Echosens, Paris, France), using the M or XL probe according to body habitus [16]. Liver stiffness was measured through an intercostal approach over the right hepatic lobe while the patient lay supine with the right arm raised. An examination was accepted when at least 10 valid measurements were obtained, and the IQR-to-median ratio was ≤30% [17]. Liver stiffness was expressed in kilopascals.

A liver stiffness measurement (LSM) of ≥8 kPa defined clinically significant fibrosis for the reference classification, in keeping with contemporary noninvasive assessment recommendations and T2DM-specific diagnostic evidence [3,17]. Transient elastography and all laboratory assessments were performed on the same day.

Sample size estimation followed a diagnostic-accuracy approach based on anticipated sensitivity and specificity, disease prevalence, confidence level, and absolute precision [18]. The formulas for sensitivity and specificity were as follows:

$n_{Sp}=\frac{Z_{1-\alpha/2}^{2}\times Sp(1-Sp)}{d^{2}\times(1-P)}$

$n_{Se}=\frac{Z_{1-\alpha/2}^{2}\times Se(1-Se)}{d^{2}\times P}$

Based on a reported fibrosis prevalence of 23.4%, sensitivity of 91.5%, specificity of 67.3%, a 95% confidence level, and 7% absolute precision in a T2DM population assessed at LSM ≥8 kPa, the sensitivity-based calculation required approximately 261 evaluable participants and was larger than the specificity-based estimate [3]. To allow for incomplete or nonevaluable assessments, the recruitment target was set at 280 patients (Figure 1).

**Figure 1:**
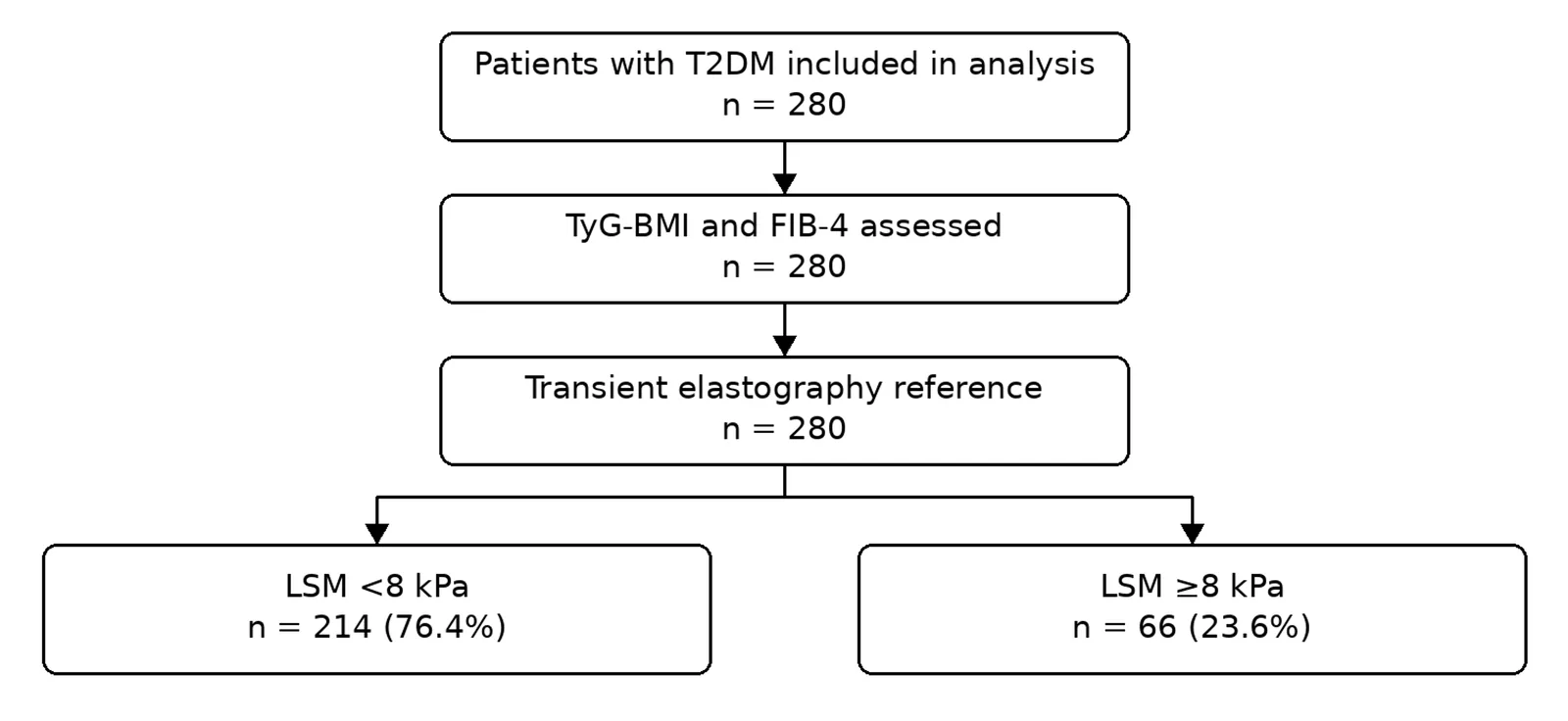
Participants included in the diagnostic analysis and classification by transient elastography (analytic population). Pre-enrollment screening and exclusion counts are not available in the analysis.

Statistical analysis was performed in IBM SPSS Statistics for Windows, version 26.0 (released 2018; IBM Corp., Armonk, NY, USA) and Python (version 3.13.5). Categorical variables were compared using the chi-square test or Fisher’s exact test, as appropriate. Continuous data were summarized as mean ± SD when approximately symmetric and as median with IQR when skewed; categorical data were summarized as n (%). Sensitivity, specificity, positive and negative predictive values, and overall accuracy were also estimated. Cutoffs for TyG-BMI and the combined model were obtained from the Youden index and regarded as exploratory; FIB-4 cutoffs were prespecified. A binary logistic regression model containing TyG-BMI and FIB-4 was fitted, and results were reported accordingly. The logistic model included an intercept, TyG-BMI per 10-unit increment, and FIB-4 per 1-unit increment, with no other predictor transformations. The exploratory combined-model probability cutoff was selected by maximizing the Youden index in the development dataset. For internal validation, participants were assigned to 10 stratified folds using a reproducible shuffled split (random seed 42); the two-predictor model was refitted in each nine-fold training set and generated predictions for the held-out fold. The pooled out-of-fold predictions were used to estimate cross-validated AUC and Brier score. Diagnostic parameters were calculated using 2 × 2 tables. All p-values were two-sided, with p ≤ 0.05 denoting statistical significance. Reporting was guided by STARD 2015 and TRIPOD+AI [19,20].

## Results

The final analysis included 280 patients with type 2 diabetes mellitus. Transient elastography identified clinically significant fibrosis (LSM ≥8 kPa) in 66 patients (23.6%; 95% CI 19.0-28.9%). Mean age was 54.3 ± 8.4 years; 152 participants (54.3%) were male, and mean BMI was 27.1 ± 3.9 kg/m². Thirty participants (10.7%) were older than 65 years, of whom 15 (50.0%) had LSM ≥8 kPa. No significant group differences were seen for sex, duration of T2DM, diastolic blood pressure, or current smoking (Table 1).

**Table 1:** Demographic and clinical characteristics according to transient elastography-defined fibrosis status (n = 280). Continuous data are shown as mean ± SD or median (25th-75th percentile), as appropriate; categorical data are shown as n (%). MD represents the mean difference for LSM ≥8 kPa minus LSM <8 kPa. OR compares the odds of the listed characteristic in the LSM ≥8 kPa group with the LSM <8 kPa group. Statistical significance was defined as p ≤ 0.05. BMI: body mass index; DBP: diastolic blood pressure; LSM: liver stiffness measurement; SBP: systolic blood pressure; T2DM: type 2 diabetes mellitus; “-”: not applicable

Variable	LSM <8 kPa, n = 214	LSM ≥8 kPa, n = 66	Test statistic	p-value	Effect estimate	95% CI
Age (years)	53.03 ± 8.09	58.44 ± 7.91	t = 4.83	<0.001	MD 5.41	3.19 to 7.62
Male sex	118 (55.1%)	34 (51.5%)	χ² = 0.27	0.605	OR 0.86	0.50 to 1.50
Duration of T2DM (years)	7.10 (5.03 to 10.97)	6.85 (4.05 to 10.15)	U = 6255	0.161	-	-
BMI (kg/m²)	26.37 ± 3.57	29.34 ± 4.02	t = 5.38	<0.001	MD 2.97	1.88 to 4.07
Obesity (BMI ≥30 kg/m²)	35 (16.4%)	28 (42.4%)	χ² = 19.66	<0.001	OR 3.77	2.05 to 6.92
Waist circumference (cm)	93.28 ± 9.66	100.09 ± 11.02	t = 4.51	<0.001	MD 6.81	3.82 to 9.80
SBP (mmHg)	127.56 ± 11.87	131.32 ± 11.04	t = 2.38	0.019	MD 3.76	0.63 to 6.90
DBP (mmHg)	77.15 ± 8.33	78.06 ± 8.13	t = 0.79	0.431	MD 0.91	-1.37 to 3.19
Hypertension	87 (40.7%)	41 (62.1%)	χ² = 9.37	0.002	OR 2.39	1.36 to 4.22
Dyslipidemia	181 (84.6%)	63 (95.5%)	-	0.02	OR 3.83	1.13 to 12.92
Current smoking	26 (12.1%)	8 (12.1%)	χ² = 0.00	0.995	OR 1.00	0.43 to 2.32

Triglycerides were higher in the fibrosis group (178.50 (142.25-222.00) vs 147.00 (113.25-194.75) mg/dL; p = 0.002), as were AST (30.00 (26.00-35.00) vs 26.00 (22.00-31.00) U/L; p < 0.001) and GGT (36.50 (29.00-43.75) vs 28.00 (21.00-40.00) U/L; p < 0.001). Platelet count was lower (203.42 ± 47.05 vs 227.78 ± 35.88 × 10⁹/L; p < 0.001). TyG-BMI and FIB-4 were also higher in patients with LSM ≥8 kPa, whereas fasting plasma glucose and HbA1c did not differ significantly (Table 2).

**Table 2:** Laboratory, metabolic, and noninvasive measures according to transient elastography-defined fibrosis status (n = 280). Continuous variables are summarized as mean ± SD or median (25th-75th percentile), according to distribution. MD is the mean difference for LSM ≥8 kPa minus LSM <8 kPa. Statistical significance was set at p ≤ 0.05. ALT: alanine aminotransferase; AST: aspartate aminotransferase; CAP: controlled attenuation parameter; FIB-4: fibrosis-4 index; FPG: fasting plasma glucose; GGT: gamma-glutamyl transferase; HbA1c: glycated hemoglobin; HDL-C: high-density lipoprotein cholesterol; LDL-C: low-density lipoprotein cholesterol; LSM: liver stiffness measurement; TyG: triglyceride-glucose; TyG-BMI: triglyceride-glucose body mass index; “-”: not applicable

Variable	LSM <8 kPa, n = 214	LSM ≥8 kPa, n = 66	Test statistic	p-value	Effect estimate	95% CI
Fasting plasma glucose (mg/dL)	160.93 ± 24.96	164.15 ± 22.66	t = 0.98	0.327	MD 3.22	-3.26 to 9.69
HbA1c (%)	8.09 ± 1.22	8.32 ± 1.12	t = 1.42	0.159	MD 0.23	-0.09 to 0.55
Triglycerides (mg/dL)	147.00 (113.25 to 194.75)	178.50 (142.25 to 222.00)	U = 8828	0.002	-	-
Total cholesterol (mg/dL)	183.52 ± 32.88	184.26 ± 34.74	t = 0.15	0.879	MD 0.74	-8.84 to 10.32
LDL-C (mg/dL)	106.58 ± 33.52	105.73 ± 38.54	t = -0.16	0.872	MD -0.85	-11.31 to 9.60
HDL-C (mg/dL)	43.91 ± 9.17	41.09 ± 9.10	t = -2.19	0.03	MD -2.82	-5.36 to -0.27
ALT (U/L)	30.50 (25.00 to 37.00)	29.00 (23.00 to 36.75)	U = 6283	0.176	-	-
AST (U/L)	26.00 (22.00 to 31.00)	30.00 (26.00 to 35.00)	U = 9332	<0.001	-	-
GGT (U/L)	28.00 (21.00 to 40.00)	36.50 (29.00 to 43.75)	U = 8996	<0.001	-	-
Platelet count (× 10⁹/L)	227.78 ± 35.88	203.42 ± 47.05	t = -3.87	<0.001	MD -24.36	-36.85 to -11.86
TyG index	9.38 ± 0.45	9.55 ± 0.47	t = 2.67	0.009	MD 0.17	0.04 to 0.30
TyG-BMI	247.62 ± 37.82	280.62 ± 43.94	t = 5.50	<0.001	MD 33.00	21.10 to 44.90
FIB-4	1.10 (0.89 to 1.40)	1.65 (1.34 to 2.11)	U = 11311	<0.001	-	-
LSM (kPa)	5.55 (4.96 to 6.19)	11.62 (10.65 to 13.01)	U = 14124	<0.001	-	-
CAP (dB/m)	232.22 ± 28.31	236.82 ± 29.34	t = 1.12	0.264	MD 4.60	-3.53 to 12.72

TyG-BMI yielded an AUC of 0.710 (95% CI 0.633-0.783). Its exploratory cutoff of 275.79 gave 54.5% sensitivity and 79.0% specificity. The AUC for FIB-4 was 0.801 (95% CI 0.736-0.857). Using FIB-4 ≥1.30, sensitivity was 78.8% and specificity was 69.2%; at ≥2.67, specificity rose to 99.5%, while sensitivity fell to 7.6%. The combined TyG-BMI + FIB-4 model had the largest AUC, 0.850 (95% CI 0.796-0.901) (Table 3).

**Table 3:** Diagnostic accuracy of TyG-BMI, FIB-4, and the combined model for clinically significant liver fibrosis. The reference classification was clinically significant fibrosis defined by LSM ≥8 kPa on transient elastography. Cutoffs for TyG-BMI and the combined model were derived with the Youden index and should be interpreted as exploratory; FIB-4 thresholds of 1.30 and 2.67 were prespecified. AUC: area under the curve; FIB-4: fibrosis-4 index; NPV: negative predictive value; PPV: positive predictive value; TyG-BMI: triglyceride-glucose body mass index

Index/model	Cutoff	Sensitivity, % (95% CI)	Specificity, % (95% CI)	PPV, % (95% CI)	NPV, % (95% CI)	Accuracy, %	AUC (95% CI)
TyG-BMI	275.79	54.5 (42.6-66.0)	79.0 (73.0-83.9)	44.4 (34.1-55.3)	84.9 (79.3-89.2)	73.2	0.710 (0.633-0.783)
FIB-4	1.3	78.8 (67.5-86.9)	69.2 (62.7-75.0)	44.1 (35.4-53.1)	91.4 (86.0-94.8)	71.4	0.801 (0.736-0.857)
FIB-4	2.67	7.6 (3.3-16.5)	99.5 (97.4-99.9)	83.3 (43.6-97.0)	77.7 (72.4-82.3)	77.9	0.801 (0.736-0.857)
TyG-BMI + FIB-4	0.234	78.8 (67.5-86.9)	79.0 (73.0-83.9)	53.6 (43.7-63.2)	92.3 (87.6-95.4)	78.9	0.850 (0.796-0.901)

The paired analysis showed an AUC gain of 0.143 for the combined model vs TyG-BMI (95% CI 0.085-0.210; p < 0.001) and 0.051 vs FIB-4 (95% CI 0.014-0.102; p = 0.002). The AUC difference between FIB-4 and TyG-BMI was 0.092 (95% CI -0.005-0.188; p = 0.062). In logistic regression, both indices contributed independently to the classification of LSM ≥8 kPa. The overall model was significant (likelihood-ratio χ²(2) = 88.38, p < 0.001) and had Nagelkerke R² = 0.407, AIC = 223.43, BIC = 234.34, and a Hosmer-Lemeshow χ²(8) = 4.41 (p = 0.818). Using the exploratory probability threshold of 0.234, the combined model identified fibrosis with a sensitivity of 78.8% and a specificity of 79.0%. The positive predictive value was 53.6%, the negative predictive value was 92.3%, and the overall accuracy was 78.9%. When this was compared with the FIB-4 cutoff of 1.30, sensitivity remained 78.8%, but specificity was higher with the combined model.

Ten-fold stratified cross-validation produced a pooled out-of-fold AUC of 0.845 (bootstrap 95% CI 0.789-0.896) and a Brier score of 0.126. The TyG-BMI cutoff of 275.788 and combined-model probability cutoff of 0.234, together with their sensitivity and specificity, represent development-sample estimates rather than separately validated cutoff performance. In the exploratory subgroup older than 65 years (n = 30; LSM ≥8 kPa, n = 15), FIB-4 ≥1.30 had a sensitivity of 100.0% (15/15) and a specificity of 60.0% (9/15). Using FIB-4 ≥2.00 in the same participants reduced sensitivity to 20.0% (3/15) and increased specificity to 93.3% (14/15). The small subgroup and the cross-sectional, elastography-based outcome limit interpretation of these age-specific estimates (Table 4).

**Table 4:** Logistic regression model combining TyG-BMI and FIB-4 for clinically significant liver fibrosis. Both prespecified index variables were entered in the model. The fitted equation was logit(p) = -10.0105 + 0.2034 × (TyG-BMI/10) + 2.4275 × FIB-4. FIB-4: fibrosis-4 index; TyG-BMI: triglyceride-glucose body mass index

Predictor variable	Regression coefficient (B)	Standard error	Wald χ² (df = 1)	p-value	Adjusted OR	95% CI
TyG-BMI (per 10-unit increment)	0.203	0.045	20.04	<0.001	1.23	1.12-1.34
FIB-4 (per 1-unit increment)	2.427	0.386	39.65	<0.001	11.33	5.32-24.12

Using the exploratory probability threshold of 0.234, the combined model identified fibrosis with a sensitivity of 78.8% and a specificity of 79.0%. The positive predictive value was 53.6%, the negative predictive value was 92.3%, and the overall accuracy was 78.9%. When this was compared with the FIB-4 cutoff of 1.30, sensitivity remained 78.8%, but specificity was higher with the combined model. This also resulted in better overall classification of patients (Figure 2).

**Figure 2:**
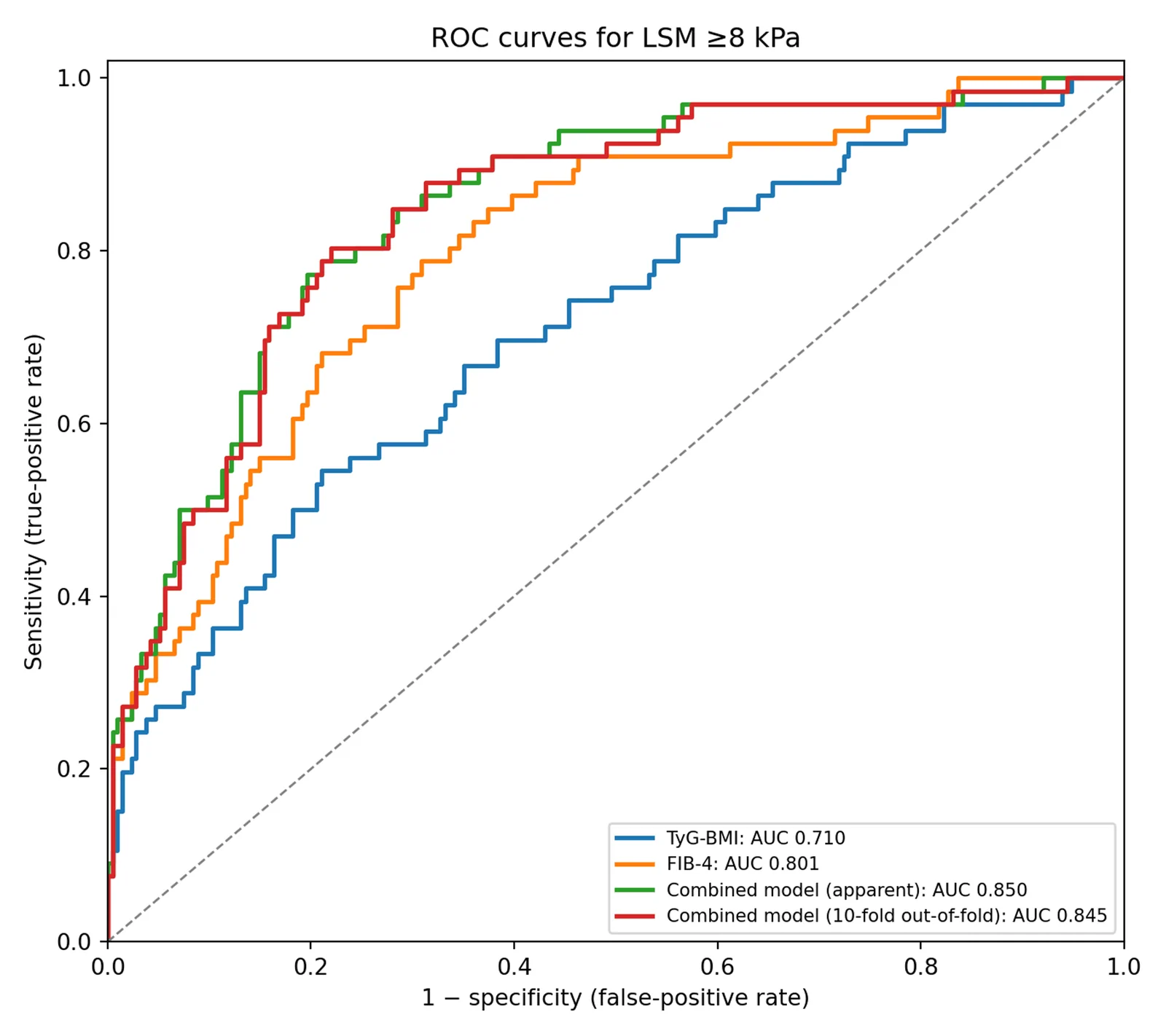
Receiver operating characteristic curves for TyG-BMI, FIB-4, the combined development model, and pooled out-of-fold predictions from 10-fold stratified cross-validation (reference standard: LSM ≥8 kPa). AUC: area under the curve; FIB-4: fibrosis-4 index; LSM: liver stiffness measurement; ROC: receiver operating characteristic; TyG-BMI: triglyceride-glucose body mass index

## Discussion

Clinically significant liver fibrosis was found in 23.6% of the patients with type 2 diabetes mellitus who were included in this study. FIB-4 had a higher AUC than TyG-BMI (0.801 vs. 0.710), although the difference was not statistically significant (p = 0.062). Combining the two indices increased the AUC to 0.850, with a statistically significant improvement over either index alone. The AUC remained similar at 0.845 after 10-fold internal cross-validation. These results indicate that combining TyG-BMI with FIB-4 may improve the identification of clinically significant liver fibrosis in patients with type 2 diabetes mellitus.

The prevalence in our study closely matched the 23.4% reported in an Indian T2DM population assessed with the same LSM ≥8 kPa threshold [3]. This agreement is plausible because both studies focused on diabetes-specific recruitment in South Asian populations and used the same elastography definition. A prevalence of 22.5% was also reported when significant fibrosis was defined as magnetic resonance elastography (MRE) ≥3.0 kPa or vibration-controlled transient elastography (VCTE) ≥8.2 kPa [2]. In that study, fewer patients met criteria for advanced fibrosis when stricter MRE ≥3.63 kPa or VCTE ≥8.8 kPa thresholds were applied [2]. By contrast, a Malaysian T2DM series reported advanced fibrosis in 22.1% despite thresholds of 9.3-9.6 kPa. Its participants had diabetes for at least 10 years and were older, which likely increased baseline fibrosis risk and may explain the high prevalence despite a stricter LSM definition [21].

TyG-BMI had an AUC of 0.710 in the present analysis. This estimate falls between the 0.729 reported in a large US MASLD sample and 0.697 reported in a Chinese MASLD population [6,8]. The Chinese study classified fibrosis from an LSM of 7.3 kPa, so inclusion of milder stiffness elevation may have reduced separation between groups [8]. A lower AUC of 0.645 was reported in a biopsy-based study [7]. Selection for biopsy and histological staging identifies a different clinical spectrum from elastography screening and may partly explain that difference. Insulin-based indices have also outperformed TyG-derived measures for fibrosis, suggesting that TyG-BMI captures metabolic burden but not all features of the fibrotic process [22]. Variation across glycemic categories further supports assessment of this index specifically in T2DM rather than direct extrapolation from broader MASLD populations [9].

FIB-4 produced an AUC of 0.801 at the 1.3 threshold, with 78.8% sensitivity, 69.2% specificity, and 91.4% NPV. In the Indian study using the same LSM threshold, specificity was similar at 67.3%, but sensitivity reached 91.5%, and the AUC was 0.901 [3]. That study showed larger between-group differences in AST and platelet count, two variables used directly in the FIB-4 formula, which may account for part of the stronger discrimination. Other T2DM studies have reported lower AUCs, including 0.653 in a sample with only 15 elastography-positive diabetic patients and 0.68 against histological advanced fibrosis [4,5]. In a Hispanic VCTE population, an AUC of 0.74 was reported, and sensitivity improved when the diabetic cutoff was lowered toward 1.0 [23]. These findings support population-specific performance rather than a single universal threshold. In our data, FIB-4 ≥2.67 was highly specific but identified few LSM-positive patients because the reference outcome began at 8 kPa and included clinically significant rather than only advanced fibrosis.

Combining TyG-BMI with FIB-4 raised the AUC from 0.710 and 0.801, respectively, to 0.850, while sensitivity remained 78.8% and specificity increased to 79.0%. A complementary effect is plausible because TyG-BMI represents glucose, triglycerides, and adiposity, whereas FIB-4 is based on age, aminotransferases, and platelet count. In South Asia, adding diabetes duration, vascular complications, and BMI to a FIB-4-based pathway reduced the number of elastography referrals, although external discrimination did not improve [10]. This shows that adding predictors is useful only when they contribute information beyond the base model. Genetic data added to FIB-4 have likewise reduced inappropriate low-risk classification [24]. Conversely, FIB-4-first pathways may miss biopsy-confirmed fibrosis, while VCTE-first strategies reduce false negatives at the cost of more hepatology referrals [25]. Current diabetes guidance therefore supports FIB-4 followed by LSM [1], and the combined model should be viewed as a potential refinement of that pathway rather than a substitute for transient elastography.

Strengths of this study include assessment of TyG-BMI and FIB-4 in the same patients, use of one transient elastography threshold throughout, a simple two-predictor model, and evaluation of discrimination, calibration, and internal validation. The study also has limitations. Its cross-sectional design addresses current fibrosis status but not future progression. Recruitment was confined to a single tertiary-care center using consecutive sampling, which may limit generalizability to other care settings and populations. Transient elastography is suitable for clinical risk assessment but does not provide histological staging and may be influenced by factors that raise liver stiffness. The TyG-BMI and combined probability cutoffs were derived from this dataset and may therefore perform less well elsewhere. Internal cross-validation addresses optimism within the sample but does not establish transportability, and PPV and NPV will change with fibrosis prevalence. Thus, the combined model showed higher apparent discrimination for LSM-defined clinically significant fibrosis in T2DM in this sample; both age-specific assessment and independent external validation are needed before clinical use.

## Conclusions

Both the TyG-BMI and FIB-4 provided useful discrimination for clinically significant liver fibrosis in patients with type 2 diabetes mellitus. FIB-4 performed better when used alone, while the combined model gave the strongest overall discrimination. The combined approach may assist preliminary noninvasive risk assessment and help identify patients who should proceed to liver stiffness testing. Because the required variables are part of routine clinical testing, the model may warrant evaluation as a triage adjunct to, rather than a replacement for, transient elastography. It should be tested in separate and more varied patient groups before it is used routinely in clinical practice.
